# Mesenchymal Stem-Cell-Derived Exosomes and MicroRNAs: Advancing Cell-Free Therapy in Systemic Sclerosis

**DOI:** 10.3390/cells14131018

**Published:** 2025-07-03

**Authors:** Cristiano Barbetta, Francesco Bonomi, Gemma Lepri, Daniel E. Furst, Silvia Bellando Randone, Serena Guiducci

**Affiliations:** 1 Division of Rheumatology, Department of Experimental and Clinical Medicine, University of Florence, AOU Careggi, 50121 Florence, Italygemma.lepri@unifi.it (G.L.); serena.guiducci@unifi.it (S.G.); 2 Department of Internal Medicine, University Hospital Careggi, 50134 Florence, Italy; francesco.bonomi@unifi.it; 3 Southern California Scleroderma and Rheumatology Centre, Los Angeles, CA 90095, USA; dan@furst.us.com

**Keywords:** mesenchymal stem cells, exosomes, microRNAs, systemic sclerosis

## Abstract

Mesenchymal stem cell (MSC) transplantation has emerged as a potential therapeutic strategy for systemic sclerosis (SSc), a rare autoimmune disease characterized by inflammation, fibrosis, and vasculopathy. Recent evidence suggests that the therapeutic benefits of MSCs do not depend directly on their ability to proliferate but rather on their capacity to release extracellular nanovesicles known as exosomes (MSC-Exos). MSC-Exos are rich in bioactive molecules such as microRNAs, which can modulate gene expression and trigger significant biological responses, playing a central role in modulating immune responses, inhibiting fibrotic pathways and promoting tissue repair and angiogenesis. Preclinical studies have demonstrated that MSC-Exos can attenuate fibrosis, modulate macrophage polarization, suppress autoreactive lymphocyte activity, and even reverse pulmonary arterial hypertension in animal models of SSc. Compared to cell-based therapies, MSC-Exos offer several advantages, including lower immunogenicity and better safety profile. This review provides an overview of the immunomodulatory, antifibrotic, and angiogenic properties of MSC-Exos and explores their potential as novel cell-free therapy for SSc.

## 1. Introduction

Systemic sclerosis (SSc) is a rare autoimmune connective tissue disease characterized by inflammation, vasculopathy, and fibroblastic dysfunction, with a high mortality rate and a significant impact on quality of life. Fibrosis of skin and internal organs is the hallmark of the disease and the main cause of morbidity and mortality [1]. Current available treatments are effective in slowing disease progression but do not offer a cure. Thanks to preclinical and human trials, many regenerative medical therapies have been studied, including mesenchymal stem cell (MSC) transplantation [2].

MSCs were first described in the 1970s and have been a focus of attention for the scientific community around the world. They are normally found in the stroma of bone marrow (BMSC), umbilical cord (hUCMSC), and adipose tissue (ASC). As multipotent stem cells, they possess the ability to grow and differentiate into chondrogenic, osteogenic, myogenic, and adipogenic lineages [3]. For these reasons, MSC transplantation has been considered as an alternative therapeutic strategy in patients with acute or chronic degenerative diseases [4].

According to scientific evidence from in vitro and preclinical studies, however, the therapeutic effects of MSCs do not depend on their proliferative or differentiation properties but, rather, on a paracrine mechanism of action. Indeed, when tissue damage occurs, MSCs reach the area of interest through the bloodstream and release biomolecular mediators with anti-inflammatory, antifibrotic, and immunoregulatory actions. In this way, they promote tissue repair and oppose possible infections. These substances (proteins, lipids, cytokines, mRNAs, and microRNAs) are not released in free form but packaged in special extracellular nanovesicles called exosomes [5,6]. Due to their ability to carry molecular loads, as well as their lower immunogenicity and tumorigenicity, MSC-Exos are attracting increasing interest as a cell-free alternative to MSC-based therapies [7]. Therefore, a detailed investigation of MSC-Exos is essential to fully exploit its therapeutic potential and safety.

Clinical studies in SSc have already shown that MSC transplantation has a beneficial effect on skin involvement. In particular, it results in a reduction in the size of ulcers and, in some cases, their complete healing with subsequent re-epithelialization of the skin. Imaging methods have confirmed increased blood flow and revascularization [8,9,10,11]. In addition, MSC transplantation in SSc has been associated with improvement of respiratory function, joint involvement, dysphagia, pericardial inflammation, and pulmonary hypertension [12,13,14,15,16]. Clinical studies have confirmed promising results in other rheumatic diseases, such as systemic lupus erythematosus, rheumatoid arthritis, Sjogren’s syndrome, dermatomyositis, and ankylosing spondylitis [17,18,19]. However, human trials evaluating MSC-derived exosome transplantation in SSc are still lacking.

The aim of this review is to depict the biological mechanisms underlying the therapeutic effects of MSC-derived exosomes in systemic sclerosis, including immunomodulatory, antifibrotic, and angiogenic properties, with a particular focus on their microRNAs cargo.

## 2. Materials and Methods

The scientific literature on the mesenchymal stem-cell-derived exosomes up to May 2025 has been considered, including both peer-reviewed non-peer-reviewed sources. The research was carried out on PubMed and EMBASE databases, searching the following keywords: “scleroderma”, “systemic sclerosis”, “mesenchymal stem cells”, “bone marrow-derived stem cells”, “adipose-derived stem cells”, “human umbilical mesenchymal stem cells”, “exosomes”, “extracellular vesicles”, “MSC-derived exosomes”, “microRNAs”, “exosomes microRNAs”, and “pulmonary hypertension”. All types of publications were considered, including original research articles, reviews, clinical trials, case reports, and experimental studies. Only English-language articles were included and abstracts without the main text were excluded.

## 3. Literature Review

### 3.1. Structure and Biological Functions of Exosomes

In nature, all prokaryotic and eukaryotic cells release extracellular vesicles. This phenomenon can be the expression of their physiological homeostasis or an abnormal condition. The study of the mechanisms underlying the formation and function of extracellular vesicles ranges from physiological regulation of tissues to pathogenic damage and organ remodeling [7].

According to MISEV2023 from the International Society for Extracellular Vesicles (ISEV), extracellular vesicle represents an umbrella definition that encompasses all cell-released and membrane-bound particles without a nucleus [20]. Extracellular vesicles are usually divided into two main categories: ectosomes and exosomes. To date, there is no definitive and universally accepted international consensus on the definition of exosomes but they are of special interest in biology because, unlike ectosomes, they do not originate directly from the budding of the cytoplasmic membrane but, rather, from an articulated process of intracellular manufacture. They contain biologically active molecules including proteins, lipids, transcription factors, DNAs, mRNAs, and microRNAs and their envelope is a phospholipid bilayer comprising cholesterol, sphingomyelin, ceramides, and tetraspanins (CD63, CD81, and CD9) [7,21]. Some proteins, such as tetraspanins, act as surface markers and they are quite heterogeneous. In particular, MSC-Exos express membrane markers common to all exosomes (CD63, CD81, and CD9) but also to the original stem cell (CD44, CD73, and CD90) [22]. For this reason, surface marker expression provides a valid strategy for their isolation and purification in vitro [23].

Exosomes’ physiological function is still partially unknown. Apparently, they play the role of biological sweepers because they maintain cellular homeostasis by removing excess substances. However, exosomes seem to go beyond the simple molecular recycling function. In fact, their content is not the result of random accumulation but rather of a targeted storage, which depends on the cell of origin, metabolic status, and external stimuli [7]. Under hypoxic conditions, for example, MSC-Exos acquire angiogenic properties and prevent tissue ischemia [24]. Once released into the extracellular environment, they merge with the membrane of a target cell and release specific mediators acting on signal transduction pathways and the gene expression profile. Therefore, exosomes appear to play an important role in intercellular communication [7].

### 3.2. MSC-Exos: Immunoregulation

**Figure 1:**
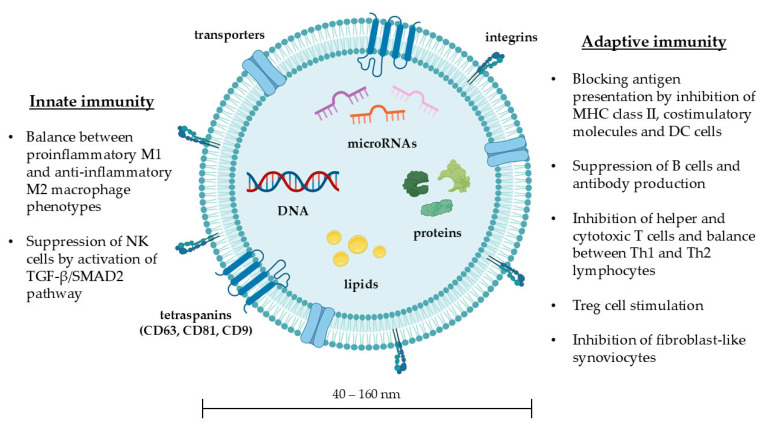
Schematic representation of an exosome, its main components and its effects on the innate and adaptive immune system.

MSC-Exos are able to regulate numerous processes involved in innate and adaptive immunity (summarized in Figure 1) [25,26,27,28,29,30,31,32,33]. First, they influence the polarization of macrophages. Depending on the biomolecular pattern of stimulation, macrophages can differentiate into a proinflammatory or an anti-inflammatory phenotype (M1 and M2, respectively). M1 macrophages release proinflammatory molecules, such as TNF-α, IL-1β, IL-6, and IL-12. In contrast, M2 macrophages release immune regulatory and profibrotic factors such as IL-4, IL-10, and IL-13 [34]. The immunomodulatory effect of MSC-Exos is closely related to the balance between M1 and M2. Studies conducted in systemic lupus erythematosus and ulcerative colitis have shown that MSC-Exos induce differentiation into M2 anti-inflammatory phenotype through regulation of the JAK1/STAT1/STAT6 signaling pathway and miR-146a release, respectively, reducing the degree of inflammation and improving survival [32,33]. In contrast, a study in BLM-treated mouse models showed that MSC-Exos stimulate differentiation into M1 proinflammatory phenotype, resulting in an antifibrotic effect [25]. Overall, MSC-Exos may promote polarization into M1 or M2 phenotype in an alternate manner, depending on the surrounding microenvironment and cytokine pattern of stimulation. This evidence is apparently conflicting but supports the hypothesis that MSC-Exos are able to regulate the balance between M1 and M2, exerting an anti-inflammatory or antifibrotic effect according to the underlying disease. Moreover, MSC-Exos inhibit proliferation, activation, and cytotoxic function of the NK cells by stimulating the TGF-β/SMAD2 transduction pathway [26].

MSC-Exos play a role in various regulatory mechanisms of adaptive immunity, including antigen presentation. They can reduce the expression of class II MHC and costimulatory molecules and, through the release of miR-21-5p, inhibit dendritic cell maturation [27]. Secondly, they can suppress lymphocyte activity. In fact, through the release of miR-155-5p, they are able to inhibit B cells proliferation, antibody production, and memory B cell development [28]. Through the release of miR-125a-3p, they suppress T cell activity, maintain balance between Th1 and Th2 lymphocytes, and inhibit the expansion of Th17 cells [29]. Through the release of miR-540-3 and miR-338-5p, they inhibit the activity of cytotoxic T lymphocytes and stimulate the proliferation of Treg cells [30]. Acting on adenosine purinergic receptors, they inhibit the activity of Th1 lymphocytes [31].

Given the ability to suppress innate and adaptive immunity, MSC-Exos transplantation has been investigated as a therapeutic strategy in preclinical models of autoimmune diseases. In Sjogren’s syndrome, MSC-Exos are able to restore the balance between Th17 and Treg cells and promote salivary secretion through miR-125b release [35,36]. In multiple sclerosis, they can cross the blood–brain barrier and promote the differentiation of microglia toward an anti-inflammatory M2 phenotype, contributing to neuroprotection [37]. In murine models of systemic lupus erythematosus, MSC-Exos have shown therapeutic potential in attenuating nephritis and diffuse alveolar hemorrhage [32,38]. In type I diabetes mellitus, they promote immunosuppression and regeneration of pancreatic islet β cells [39,40]. Moreover, experimental models of rheumatoid arthritis have demonstrated that exosomal delivery of microRNAs (such as miR-451a, miR-205-5p, miR-150-5p, and miR-320a) inhibits the expansion of fibroblast-like synoviocytes and prevents synovial hyperplasia [41,42,43,44]. Finally, promising results have been reported in preclinical studies on skin psoriasis, uveitis, and inflammatory bowel disease [45,46,47].

Overall, MSC-derived exosomes exert multifaceted effects on both innate and adaptive immune responses. By restoring immune balance and promoting immunological tolerance, MSC-Exos confirm therapeutic effects in experimental models of autoimmune diseases. These properties appear particularly relevant to systemic sclerosis, where immune dysregulation plays a central role in disease pathogenesis.

### 3.3. MSC-Exos’ Antifibrotic Properties

Subcutaneous injection of bleomycin (BLM) causes abnormal deposition of extracellular matrix and partially mimics the typical skin fibrosis of SSc. Therefore, the BLM-treated mouse model is widely used to investigate possible novel treatment [25,48,49,50]. Preclinical studies have shown that MSC-Exos transplantation is an effective therapeutic strategy due to anti-inflammatory, antifibrotic, and angiogenic effects [25,48,49,50,51,52,53]. Specifically, in the studies by Yu et al. [25] and Jin et al. [48], BLM-treated mice were transplanted with human umbilical cord mesenchymal stem cell exosomes (hUCMSC-Exos) and murine bone marrow mesenchymal stem cell exosomes (BMSC-Exos), respectively, with very similar results (Table 1). Macroscopically, the BLM group showed a thickened skin layer, while the MSC-Exos group showed normal thickness. Histologically, MSC-Exos group showed a significant reduction in inflammatory infiltrate, ECM deposition, and hydroxyproline content compared to the BLM group. Molecular analysis methods confirmed the reduced expression of tissue fibrosis markers such as TGF-β1, collagen 1, and fibronectin 1. These data indicate that MSC-Exos can effectively counteract BLM-induced fibrosis [25,48].

The antifibrotic power of MSC-Exos was further confirmed by immunohistochemical analysis on epithelial–mesenchymal transition (EMT), a peculiar phenomenon involved in the pathogenesis of SSc (summarized in Table 1). During specific stimulation, skin epithelial cells can detach from adjacent cells, acquire mesenchymal characteristics, and differentiate into myofibroblasts [54]. BLM-treated mice exhibited high levels of vimentin and α-SMA, indicating pronounced epithelial–mesenchymal transition and fibroblastic activity. Conversely, mice treated with MSC-Exos displayed reduced expression of these biomarkers, underscoring the ability of MSC-Exos to inhibit the EMT process [25].

### 3.4. Exosomal Content: microRNAs

It is known that, although non-coding nucleotides, microRNAs are not simply “junk” molecules but perform regulatory and intercellular communication functions. In fact, through base pairing, they modulate the expression of the human genome [55]. They are involved in the pathogenesis of several autoimmune diseases, such as type I diabetes mellitus [56,57], systemic lupus erythematosus [58,59,60], and multiple sclerosis [61,62]. Notably, miR-618 appears to be overexpressed in SSc patients. By binding to IRF8 mRNA, it promotes IFN-α release and inhibits plasmacytoid dendritic cell development [63]. Similarly, other isoforms such as miR-203 3p, miR-155, miR-130b, and miR-483-5p promote fibroblast activation by targeting matrix metalloproteinase 1 (MMP1) and peroxisome proliferator activated receptor γ (PPARγ) [64,65,66,67].

MSC-Exos contain a broad spectrum of proteins and nucleic acids. Proteomic analyses have identified about 730 proteins regulating cell growth, proliferation, adhesion, migration, and morphogenesis [68]. On the other hand, MSC-Exos contain a heterogeneous pool of microRNAs. For this reason, isolating the single molecule and quantifying its contribution to the overall phenotypic response is quite difficult. The study by Ferguson et al. [69] identified the top 23 microRNAs, accounting for 79.1% of total microRNAs content. The remaining microRNAs were considered to have a marginal contribution to the final biological effect and were hence excluded from further analysis. Subsequently, target genes were traced, resulting in the regulation of three main processes: angiogenesis, cell growth, and fibrosis (summarized in Table 2).

MiR-21, miR-23a-3p, miR-424-5p, miR-144, and miR-130a-3p target genes are involved in angiogenesis. In particular, they enhance proteins involved in VEGF pathway (such as angiopoietin, angiopoietin receptor, and neuropilin 2) and FGF pathway (such as transcription factor STAT5A). They also silence genes coding for angiogenesis inhibitors [69]. 

Several microRNAs regulate over 60 genes involved in cell growth and death, such as *EGFR*, *PI3K*, and *p53*, thereby promoting cardiomyocytes proliferation [69].

All the top 23 microRNAs target genes involved in the TGF-β, Wnt and PDGF signaling pathways. These mediators promote fibroblastic differentiation and deposition of extracellular matrix components, such as collagen and fibronectin. Some microRNAs such as miR-29 and Let-7 directly target collagen and fibrillin-1 genes (as *COL1A1*) [69].

### 3.5. Exosomal microRNAs’ Antifibrotic Properties

**Figure 2:**
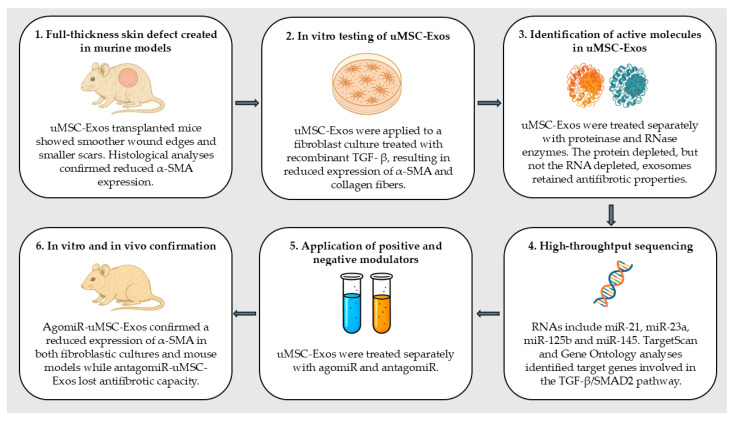
Key steps in the study by S. Fang et al.: transplantation of hUMSC-Exos on murine models with skin defect [70].

MSC-Exos transplantation effectively limits scar formation in animal models [49,50,51,53,70,71]. S. Fang et al. [70] showed that mice with a skin defect undergoing MSC-Exos transplantation had wounds with smoother edges, smaller scars, and reduced α-SMA expression compared with control. In vitro tests confirmed these results. By alternative application of degradative enzymes (proteinases and RNases) and ribonucleotide expression modulators (microRNA agonists and antagonists), it was established that the antifibrotic effect does not depend on their protein content but rather on the presence of microRNAs, such as miR-21, miR-23a, miR-125b, and miR-145, that suppress the differentiation into myofibroblasts by inhibiting the TGF-β/SMAD2 signaling pathway. Interestingly, most of these microRNAs are highly expressed in MSC-Exos but not equally expressed in the original cell, suggesting they are actively stored in exosomes as biological mediators. The main steps of the study are summarized in Figure 2 [70].

It was confirmed that miR-214 is under-expressed in patients with SSc, suggesting a possible role as a disease biomarker [72]. On the other hand, application of BMSC-Exos in cell cultures was associated with increased miR-214 expression and reduced fibroblastic activity, while addition of miR-214 inhibitor was associated with increased fibroblastic proliferation. Apparently, BMSC-Exos are able to deliver miR-214 within fibroblasts, down-regulate the IL33/ST2 signaling pathway and thereby reduce their growth, migration, and gene expression [51].

Moreover, MSC-Exos from human adipose tissue and mouse models contain miR-29a-3p, which inhibits fibrosis of the skin and lungs, as confirmed by the reduction in TGF-β1 and α-SMA [49].

### 3.6. MSC-Exos and microRNAs: Pulmonary Hypertension

Several studies on mouse models have shown that MSC-Exos possess the ability to slow down and even reverse pulmonary arterial hypertension (PAH), a common feature in many rheumatic diseases [73,74,75,76]. The pathogenetic mechanism involves an imbalance between vasoconstrictors, such as endothelin-1, and vasodilators, such as nitric oxide and prostaglandins. Vascular endothelium dysfunction and smooth muscle hypertrophy lead to remodeling of small- and medium-sized arteries. These alterations, together with any concomitant interstitial lung disease, promote an increase in vascular resistance and, consequently, an increase in mean pulmonary pressure [77].

Environmental or biochemical stimuli may promote smooth muscle proliferation in the pulmonary arteries by phosphorylation of STAT3 transcription factor. Hypoxia, moreover, can lead to macrophage activation and inflammation of the lung parenchyma [74]. MSC transplantation is able to inhibit macrophage infiltration, extinguish lung inflammation, suppress vascular remodeling, and reduce mean pulmonary pressure, preventing PAH. Indeed, MSC-Exos inhibit STAT3 activity and modify microRNAs expression. For example, they induce overexpression of miR-204, which is usually reduced in PAH, and silencing of miR-17, which is usually increased in pulmonary hypoxia. As a secondary effect, MSC-Exos suppress the release of inflammatory molecules such as MCP-1, HIF, and IL-6. Macroscopically, they prevent vascular remodeling and inhibit hypertrophy of smooth muscle tissue, as confirmed by low levels of α-SMA [74].

Studies on mouse models of PAH have shown that MSC-Exos not only prevent the increase in lung pressure but can also reverse the process once it is started [75,76]. Indeed, they inhibit apoptosis of endothelial cells and proliferation of smooth muscle cells in the pulmonary arteries. They stimulate the formation of capillary-like structures by endothelial cells in vitro, supporting their neoangiogenic potential. They suppress macrophage infiltration and EMT, which is responsible for differentiation into myofibroblasts. As a result, they reduce fibrosis, pulmonary artery thickness, and right ventricular hypertrophy [75,76]. Apparently, the anti-inflammatory and anti-proliferative properties of MSC-Exos are mediated by the overexpression of the Wnt5a protein in hypoxic pulmonary vascular cells and by the release of microRNAs such as miR-34a, miR-122, miR-124, and miR-127 [75,76].

Based on this evidence, exosomes seem to play a key role in the vascular remodeling of pulmonary circulation suggesting the potential application of MSC-Exos transplantation as a cell-free therapy in PAH related to SSc or other connective tissue disorders.

## 4. Discussion: MSC-Exos Transplantation as a Cell-Free Therapy

Although MSCs have demonstrated a beneficial effect in numerous diseases, they are not considered a first-line therapy. Their use is still associated with unresolved risks. Adverse reactions range from minor events such as upper respiratory infections, dizziness, and diarrhea to serious complications such as osteomyelitis, ventricular arrhythmia, iatrogenic carcinogenesis, immune-mediated rejection, infusion toxicity, and rarely death [9,13,14,15,16,78,79]. MSC limitations are mostly related to their cellular nature. First of all, due to their large size, they are blockaded in the pulmonary capillaries as soon as they reach microcirculation [5]. Thus, intravenous administration is associated with the risk of thrombosis and pulmonary embolism [80]. Secondly, chromosomal abnormalities may occur, resulting in growth of malignant tumors [81]. Moreover, they age rapidly and their production is particularly expensive [82].

On the other hand, exosomes have been shown to play a key role in the mechanism of MSCs. First, they are easier to store, analyze, and produce on a large scale [5,82,83]. Freezing and thawing does not affect their number, size, or marker expression [83]. They are associated with a lower risk of thromboembolic events because, thanks to their nanometric size, they can pass freely through biological barriers without compromising microcirculation. They can reach long distances, penetrate tissues effectively, and deliver their content into target cells [84]. Exosome microRNAs are more stable than cellular microRNAs and can resist ribonuclease-induced degradation [84]. While MSC transplantation is followed by a rapid cell decline in vivo, exosomes remain active and have more lasting effects. They are associated with a lower risk of carcinogenesis because they do not replicate. Compared to MSCs, exosomes are considered well-tolerated, non-toxic, and non-immunogenic. Indeed, rejection after plasma or blood transfusions is not due to immune reactions against exosomes [7]. In addition, administration of MSC-derived extracellular vesicles is considered safe and may improve renal function in patients with chronic kidney disease [85].

MSC-derived extracellular vesicles have already been tested in a clinical case of a 55-year-old woman with SSc-related interstitial lung disease, resulting in marked improvement of clinical symptoms, imaging findings, exercise tolerance, and supplemental oxygen need [86]. However, substantial gaps remain before MSC-Exos can be translated into clinical therapy for SSc. Unfortunately, current research is limited by difficulties of visualization, isolation, and detection of single exosomes, both in vitro and in vivo. Purification by centrifugation has not yet been perfected, as it rather results in a heterogeneous pool of extracellular vesicles. A chromatographic isolation method based on expression of surface markers such as tetraspanins (CD63 and CD81) has been proposed for this purpose [87]. However, there is still no isolation method designated as a “gold standard”. Molecular analyses require the purification of many exosomes and, unfortunately, they do not always contain sufficient amounts of microRNAs [88]. Although bioreactor cultures have been tested to increase the number of exosomes, large-scale production for clinical use remains a challenge and calculation of production rate is difficult because exosomes are continuously released and picked up by cells in a dynamic process [7,83]. Moreover, exosomes possess a short biological half-life due to renal, hepatic, and pulmonary clearance [89].

With regard to promising prospects, hydrogel has recently attracted attention as a biocompatible auxiliary substance. Indeed, hydrogel injection has been shown to prolong the half-life of exosomes in experimental studies on ischemia and prevention of scar hyperplasia [90,91]. In addition, MSC preconditioning with specific stimulation may pave the way for future applications. In a preclinical study of SSc, pretreatment with IFNγ resulted in exosomes with enhanced antifibrotic potency [92]. Moreover, biomolecular programming techniques can reproduce in vitro expression of surface markers in order to direct exosomes towards specific target cells. Similarly, they can manipulate the content by inserting specific substances such as antisense oligonucleotides, chemotherapeutic agents, and immunomodulators. For instance, electroporation does not require chemical reagents or viral particles and, as such, poses less risk to patients [51,69,83]. Bioengineered MSC-Exos have already been created to balance the M1/M2 polarization and suppress joint inflammation in murine models of rheumatoid arthritis through the delivery of let-7b-5p and miR-24-3p [93]. Similarly, genetically engineered cellular nanovesicles bearing CD40 were found to effectively inhibit the immune response in mouse models of systemic lupus erythematosus and especially deliver a mycophenolate load by achieving an enhanced outcome [94]. Such evidence supports the application of MSC-Exos as possible nanoscale pharmacological vehicles. Furthermore, engineered exosomes carrying non-coding RNAs such as small-interfering RNAs (siRNAs) or miRNAs have already been designed for treatment of neoplastic and neurodegenerative diseases [7,83], while exosomes loaded with miR-199a or miR130a-3p have been shown to stimulate cardiomyocyte proliferation in a dose-dependent manner. Indeed, highly loaded exosomes possess greater anti-apoptotic properties than native or low-dose loaded exosomes [69]. Thus, processing exosomes with a higher content of microRNAs would allow the overall therapeutic effect to be enhanced. Finally, identification of single exosomes by cryoelectron microscopy may improve our understanding of their biology and use in applied science [7].

Key areas for future investigations should include the standardization of isolation methods, analysis protocols, and dosing strategies to improve reproducibility, consistency, and safety across studies [5]. Any clinical trial on SSc should prioritize endpoints such as improvement in skin thickening, digital ulcers, and pulmonary arterial hypertension, as well as patient-reported quality-of-life outcomes. Route and dose of administration should be optimized to ensure patient safety. Indeed, pharmacokinetic properties of exosomes depend on their lipid and protein composition [7]. Moreover, long-term immunomodulatory effects and persistence of therapeutic benefits require further elucidation [5].

## 5. Conclusions

In recent years, MSC-Exos have gained increasing attention as a potential cell-free therapeutic strategy, especially in SSc. In preclinical BLM-mice models of SSc, they have shown efficacy in reducing inflammation, fibrosis, and pulmonary arterial hypertension. Available evidence suggests that immunomodulatory, antifibrotic, and pro-angiogenic effects depend on the release of functional microRNAs capable of targeting key molecular pathways, including TGF-β/SMAD, Wnt, and IL-33/ST2. Compared to stem cell transplantation, exosomes offer significant advantages in terms of safety, stability, immunogenicity, and potential for in vitro engineering.

While preclinical results are promising, current evidence is limited to in vitro and animal models, with a significant lack of clinical trials evaluating safety and efficacy in humans. This gap highlights the need for well-designed clinical studies to validate these findings and to establish standardized protocols for exosome isolation, characterization, and delivery.

In conclusion, this review offers an integrated perspective on the immunomodulatory, antifibrotic, and angiogenic properties of MSC-Exos as potential cell-free therapy in SSc, with a particular emphasis on their microRNA cargo. By critically assessing both mechanistic and translational aspects, this review aims to guide future research priorities and clinical development in the field.

## Figures and Tables

**Table 1:** Summary of findings from Yu et al. and Jiahui et al., in which bleomycin-treated mice (BLM group) received subsequent transplantation with mesenchymal stem-cell-derived exosomes (MSC-Exos group). Differences between the two groups are observed from a macroscopic, histological, and molecular point of view [25,48].

Differences	BLM Group	MSC-Exos Group	Reference
Macroscopic	Thickened skin layer, reduced subcutaneous adipose tissue	Skin and subcutaneous tissue of normal thickness	[25,48]
Histological	Macrophages and CD4+/CD8+ T lymphocytes inflammatory infiltrate, abundant ECM, collagen fibers and hydroxyproline	Significant reduction in inflammatory infiltrate, collagen fiber deposition and hydroxyproline content	[25,48]
Molecular	High levels of tissue fibrosis markers (TGF-β1, collagen 1, and fibronectin 1)	Reduced tissue fibrosis markers (TGF-β1, collagen 1, and fibronectin 1)	[25,48]
EMT	High levels of vimentin and α-SMA (high differentiation of endothelial cells into mesenchymal cells with fibroblastic activity)	Low values of fibroblast markers (vimentin and α-SMA), suggesting inhibition of the EMT process	[25]

**Table 2:** Overview of the most common MSC-Exos microRNAs, corresponding target pathways, and biological effects.

microRNAs	Target Pathways	Biologic Effect	Reference
miR-214	IL-33/ST2	Alleviates skin fibrosis in systemic sclerosis by blocking the IL33/ST2 axis	[51]
miR-23a-3p	P53, PI3K	Cell cycle regulation	[69]
miR-424-5p	VEGF	Angiogenesis stimulation	[69]
miR-144-3p	PDGF	Cell survival, anti-apoptosis	[69]
miR-130a-3p	HOXA5	Angiogenesis stimulation	[69]
miR-145-5p	TGF-β	Inhibition of fibroblast proliferation	[69]
miR-29a/b-3p	COL1A1, FBN1	Reduction in collagen synthesis	[69]
miR-221-5p	Wnt	Endothelial proliferation	[69]
miR-21-5p	Crim1	Cardiomyocyte survival	[69]
miR-125b-5p	TP53, BAK1	Apoptosis inhibition	[69]
miR-22-3p	TGF-β, PI3K	Protection from oxidative stress	[69]
miR-199a-3p	ADAMTS3, p53	Regulation of energy metabolism	[69]
miR-191-5p	BDNF	Cellular differentiation	[69]
let-7a/b/i	COL1A1, HMGA2	Reduction in fibroblast proliferation	[69]
miR21,miR-125b, miR-145	TGFβ/SMAD2 pathway	Suppression of myofibroblast differentiation, Reduction in scar formation	[70]
miR-196b-5p	COL1A2	Reduction in collagen synthesis	[71]

## Data Availability

No new data were created or analyzed in this study. Data sharing is not applicable to this article.

## Abbreviations

The following abbreviations are used in this manuscript:

ASC	Adipose-derived stem cell
BLM	Bleomycin
BMSC	Bone-Marrow-derived Mesenchymal Stem Cell
ECM	Extracellular Matrix
EGFR	Epidermal Growth Factor Receptor
EMT	Epithelial–Mesenchymal Transition
HIF	Hypoxia-Inducible Factor
hUCMSC	human Umbilical Cord Mesenchymal Stem Cell
IFN	Interferon
IRF	Interferon Regulatory Factor
IL ISEV	Interleukin International Society for Extracellular Vesicles
FGF	Fibroblast Growth Factor
MHC	Major Histocompatibility Complex
miR	microRNA
MCP-1 MISEV	Monocyte Chemotactic Protein-1 Minimal Information for Studies of Extracellular Vesicles
MMP	Matrix Metalloproteinase
MSC MSC-Exos	Mesenchymal stem cell Mesenchymal stem-cell-derived exosomes
NK	Natural Killer
PAH	Pulmonary Arterial Hypertension
PDGF	Platelet-Derived Growth Factor
PI3K	Phosphoinositide 3 kinases
PPARγ	Peroxisome Proliferator Activated Receptor γ
SMA	Smooth Muscle Actin
SMAD	Small Mother Against Decapentaplegic
SSc	Systemic sclerosis
STAT	Signal Transducer and Activator of Transcription
TGF	Transforming Growth Factor
Th	T helper
TNF	Tumor Necrosis Factor
Treg	T regulatory
VEGF	Vascular Endothelial Growth Factor
